# Some Observations upon Pelvic Cellulitis

**Published:** 1887-10

**Authors:** Virgil O. Hardon

**Affiliations:** Professor of Obstetrics and Diseases of Women and Children, Atlanta Medical College, Atlanta, Ga.


THE ATLANTA MEDICAL AND SURGICAL JOURNAL.

VOL. IV. OCTOBER, 1887. No. 8.

### Original Communications.

### SOME OBSERVATIONS UPON PELVIC CELLULITIS.

(Read before the Medical Association of Georgia, April 22, 1887.)

BY VIRGIL O. HARDON, M. D., PROFESSOR OF OBSTETRICS AND DISEASES OF WOMEN AND CHILDREN, ATLANTA MEDICAL COLLEGE, ATLANTA, GA.

I propose in this paper to offer some views upon the subject of pelvic cellulitis from the stand-point of my own experience and observation. In presenting these views I desire to say in advance that they are offered less with the purpose of insisting upon their correctness than with the aim of subjecting them to criticism and experiment at the hands of fellow-practitioners. Since these views have grown out of clinical phenomena which have presented themselves in my own practice, I shall endeavor to fortify them by reports from my case-book, and thus to avoid the charge of theorizing and of *a priori* reasoning.

### ACUTE PELVIC CELLULITIS.

CASE I.—On the 21st of January, 1887, I was requested by Dr. N. O. Harris, of this city, to see with him Minnie L., a prostitute twenty-four years of age, who had borne a child three months before. The history of the patient showed that her menses had always been of normal character up to her pregnancy, and that she had never suffered from any symptoms which would indicate pelvic disease of any kind. Since her labor she had had tenderness of the abdomen and pain in walking and in micturition. Her general health, however, had been good. On the day before I saw her she was seized with pain in the back, pelvis, hips, abdomen and thighs. This pain was acute and excessive. Micturition and defecation became very painful, especially the latter. She had a slight chill, followed by high fever, thirst and complete loss of appetite. When seen by me she was in bed, tossing and moaning with pain, which was referred principally to the pelvic region. Pulse 120, temperature 101, skin hot and dry, face flushed, tongue coated. Vaginal and rectal examination were rendered impossible by excessive tenderness of the parts. The following morning she was fully anæsthetized and a complete examination effected. The vagina was hot and dry. The cervix was lacerated on the left side. The womb was low in the pelvis and was pushed forward against the bladder. In the posterior fornix, and occupying the whole space between the cervix and the rectum, could be felt a rounded, bulging mass which had a boggy, œdematous feeling. By a finger in the rectum this mass could be outlined and felt to extend upward about an inch. No fluctuation could be detected, and when pressed by the finger the mass could not be displaced upward. Considering the condition to be that of pelvic cellulitis in the stage of serous infiltration, I decided to attempt to draw off the serum from the cellular tissue, hoping thereby to abort the disease and prevent the formation of solid plastic exudation with possibly a subsequent abscess. Accordingly an aspirator needle was thrust into the tumor from the vagina at three different points successively and about an ounce in all of serum tinged with blood was withdrawn. The tumor was then found to be so softened and diminished in size as to be scarcely perceptible to the touch. A quarter grain of morphine was given hypodermically, and the patient ordered to remain perfectly quiet in bed and take only liquid diet. When seen twenty-four hours later she had had a good night's sleep, the pain in the pelvis was almost entirely gone, defecation was no longer painful, appetite had returned, the pulse had fallen to 80, the temperature to 99, and the patient begged to be allowed to get up. The mass in the posterior fornix could be felt only as a slight thickening. Two days later the patient was apparently in her usual health.

From a clinical point of view there are three stages in the progress of an attack of acute pelvic cellulitis, viz., the stage of serous infiltration, the stage of solidification and the stage of suppuration. Under favorable circumstances a stage of absorption takes the place of the stage of suppuration and the patient is spared the suffering and danger involved in the formation of an abscess. The stage of serous infiltration is a brief one, lasting usually not more than forty-eight hours. Hence it often happens that the patient is not seen by the physician until this stage has passed. The most prominent physical feature of this stage is the infiltration of the cellular tissue with serum, so that it is in an œdematous condition and presents to the finger what has been described as a boggy or doughy feeling, which is characteristic of œdema and is readily recognized by the educated touch. This infiltration of serum is a consequence of arterial congestion and the transudation through the walls of the blood-vessels might be said to be nature's method of relieving such congestion. As soon as this transudation takes place the arterial tension is lessened, or, in other words, the congestion is diminished, and the effect of such diminution is shown by a decline of the pulse and temperature and a general abatement of the constitutional symptoms. The progress of the disease would probably be arrested at this point were it not for the fact that the infiltrated serum, inclosed within the meshes of the cellular tissue, soon becomes solidified and is converted into so-called plastic exudation which acts as a foreign and irritating substance and is gotten rid of only by the tedious process of absorption or the dangerous process of suppuration.

An obvious indication then during the stage of serous infiltration is to get rid of the effused serum before it has undergone solidification and thus to arrest the disease at that point. Can this be accomplished? Emmet recommends for this purpose vaginal injections of hot water, which he says "should be continued literally *for hours*, if possible, and be repeated at short intervals." He further adds, in his characteristic positive style, "It is the only means we possess for aborting an attack of cellulitis, which *it will do if thoroughly employed at the beginning*."[1] (The italics are Emmet's). I am prepared to indorse this recommendation of Emmet's, but I must decline to admit that the method is infallible, or that all other methods are futile. The vaginal douche of hot water is unquestionably a very efficient promoter of absorption. But when the effused serum is large in quantity the absorbent vessels are sometimes apparently unequal to the task of disposing of so extensive an accumulation.

As a result of my own experience, I feel justified in claiming that the mechanical removal of the infiltrating fluid by aspiration offers a valuable addition to our resources in such cases. My method of procedure is to introduce the aspirator needle into the infiltrated cellular tissue and to maintain a constant suction until the serum ceases to flow. I then withdraw the needle and introduce it at another point and repeat the suction as before. By continuing these punctures at various points the distention will gradually be diminished and the tissues will be reduced very nearly to their normal condition. The pain will be relieved, the febrile action will subside, and the patient will progress to a rapid convalescence. The disease is thus effectually aborted and does not go on to the stage of solidification, and hence the tedious process of absorption and the danger of suppuration are avoided.

When the effusion has taken place into the broad ligament on one or both sides of the cervix, I have found that some care in aspirating is necessary in order to avoid wounding the uterine artery. If deep pressure be made the pulsation of this artery may usually be detected by the finger, and thus the danger of its injury may be avoided. The punctures may safely be made to the depth of a half an inch or even more, since the peritoneum

[1]Principles and Practice of Gynæcology, New York, 1884, p. 261.

is pressed upward toward the abdominal cavity as much as the mucous membrane is pressed downward into the vagina, and hence the thickness of the cellular tissue at the point of infiltration is much greater than in its normal condition. Emmet says: "I cannot regard the introduction of the trocar into the inflamed tissues of the pelvis as a procedure free from danger under all circumstances."[1] As far as my experience goes, I am satisfied that this statement is not true of the introduction of the aspirator needle into the infiltrated pelvic cellular tissue.

My experience with this method of treatment has been confined to a small number of cases, and I would feel a diffidence in presenting it to the profession were it not that the results in the few instances in which it has been used have been so eminently satisfactory. As far as I am aware the method has never been made use of in any other hands than my own, as I find no allusion to it in the gynæcological literature at my disposal. Brickell, of New Orleans, published an article ten years ago,[2] in which he described a form of pelvic cellulitis in which the inflammatory process terminated in the formation of permanent collections of serum within the pelvic cellular tissue and advocated the evacuation of such collections by means of the aspirator. But this condition is quite different from that to which I refer. It is only to the first stage of those cases which, if let alone, would go on to solidification that my method is applicable.

The following case illustrates the failure of the vaginal douche of hot water to cause absorption of the infiltrated serum when the infiltration involves a large extent of tissue.

CASE II.—E. G., colored, married, age 26, was delivered January 16, 1886, of her sixth child after an easy labor. All went well until the morning of the ninth day, when she got up and sat in her stocking feet by the fire. Late in the afternoon she was seized with a chill, and had severe pain in the pelvis, abdomen and back. I saw her the following morning, when she complained of severe pain, tenderness of the abdomen, thirst, anorexia, and

[1] Op. cit., p. 270.

[2] "The Proper Treatment of Pelvic Effusions," Amer. Journal of Medical Sciences, April, 1877.

intense headache and nausea. Defecation and micturition gave intense pain. Pulse 140, temperature 102 2-5. By vaginal examination the cellular tissue on each side of and behind the cervix was found to be swollen, bulging into the vagina and presenting the characteristic boggy feeling of acute cellulitis. The patient was ordered to remain in bed, was given a quarter grain of morphine hypodermically, and was directed to use a copious hot vaginal douche of not less than two gallons every two hours. She possessed unusual intelligence for a negro, and I satisfied myself that my directions were faithfully carried out. This free use of hot water was kept up for forty-eight hours. At the end of that time solidification had taken place and the womb had become immovably fixed in a hard mass of plastic exudation which surrounded it on three sides and pushed it forward against the bladder. Suppuration subsequently took place, the abscess discharging through the vagina, and the patient recovered only after a long and exhausting illness.

In this case I consider that the amount of serum was so great that the pelvic absorbent vessels were unable to remove it. Had the tissues been punctured at various points, as in Case I, and the serum withdrawn by aspiration, I believe that the disease might have been aborted. Such would be my treatment of a similar case at the present time.

### CHRONIC PELVIC CELLULITIS.

CASE III.—A. C., white, married, age 36, has had one miscarriage and five children at term, the last one six years ago, since which time she has been in bad health. Five years ago she had perineorraphy performed in New Orleans. She experienced some relief from the operation, but still remained an invalid. The last menstrual period was delayed two weeks beyond the usual time. As she had always been regular, she attributed the delay to a severe cold from which she suffered at the time. The flow was accompanied by excessive pain and by high fever, loss of appetite, thirst, great tenderness in the abdomen and scanty and painful micturition.

I was requested by Dr. D. H. Howell, of Atlanta, to see this patient, November 23, 1885. Examination revealed great tenderness throughout the vagina. The cervix was about an inch from the vulva, pointing in the direction of the vaginal axis. It was lacerated on both sides and the lips were everted. The body was retroflexed, pressing against the rectum, and was tender, enlarged and congested. The cervix was surrounded and held in its position of prolapse by a firm deposit in the cellular tissue on all sides, exceedingly tender to the touch. The womb was absolutely immovable. The sound passed backward at an acute angle. There was no prolapse of bladder or rectum.

The results of physical examination, taken in connection with the history of the case, made the diagnosis easy. I had to do with a case of acute pelvic cellulitis in the stage of solidification. The cervix and the whole vaginal roof were painted with compound tincture of iodine twice a week, a pledget of cotton saturated with glycerine was inserted in the vagina daily, and the copious vaginal douche of hot water was directed to be used night and morning. This treatment was continued for two months, except during the menstrual period. The hardness and tenderness about the womb gradually diminished and the organ became movable. On the 15th of January the womb could be restored to its normal position without pain. All the tenderness and congestion had ceased, and the patient had improved greatly in appetite, strength and flesh.

On the 26th of January, five days after the cessation of the menses, with the assistance of Drs. Harris and Wile, the patient was etherized and trachelorraphy performed. Perfect union resulted, and since that time the patient has enjoyed robust health.

CASE IV.—A. M., white, married, age 34, has been gradually failing in health since the birth of her last child two years ago. Has lost flesh, strength and appetite without any apparent reason. Has bearing-down pains and painful locomotion. Her greatest suffering, however, is from frequent micturition. She is obliged to urinate about every hour during the day and to rise several times during the night for this purpose. This symptom is aggravated at the menstrual periods.

Examination made February 23, 1887, showed the womb to be low down in the pelvis, but perfectly movable. The cervix was lacerated bilaterally and the lips everted. On each side of the cervix, in the cellular tissue of the broad ligaments, could be felt two hard, irregular masses about the size of an almond, which were tender to the touch. There were no indications of any disease of the bladder or urethra, and the urine showed nothing abnormal by chemical or microscopical examination.

The womb was restored to its normal position and its normal axis in the pelvis, and held in that position by the supporting tampon, consisting of a ring of pledgets of cotton encircling the cervix and a larger underlying pad of cotton lifting it from the posterior vaginal wall. The patient was sensible of immediate relief of the bearing-down pain which she had previously experienced. This supporting tampon was renewed every second day. No other treatment was used except a laxative pill occasionally. This course was pursued up to the next menstrual period, which occurred March 13th.

On the night after the first tampon was applied, the patient was obliged to rise once to urinate. After that she never rose in the night as long as the tampon was worn. The bearing-down feeling, the pain and the difficulty of locomotion rapidly diminished, and before the end of ten days had ceased altogether. The hard and tender masses on each side of the cervix were sensibly diminished at the second dressing, and at the fourth dressing on the seventh day could no longer be felt.

Upon the appearance of the menses the patient came to Atlanta for the purpose of submitting to the operation of trachelorraphy, which had been represented to her as the *ultimatum* of the treatment. But at the last moment her courage failed her. She declined to have the operation performed and returned to her home. I therefore resigned all connection with the case. I have met her frequently since, and she informs me that all her old symptoms have returned.

These two patients furnish typical illustrations of two classes of cases which are constantly coming under my observation, which present many points of similarity upon superficial examination, yet which experience has taught me to differentiate. As I find no distinction between these two groups in gynæcological literature, I am led to believe that such distinction is generally overlooked, and I believe further that a recognition of it will add much to the success with which such cases can be treated.

The teachings of Emmet, the apostle of pelvic cellulitis, are that as long as there are hardness and tenderness in the vaginal roof, these two phenomena furnish sufficient evidence of the existence of chronic pelvic cellulitis, and that during the persistence of these symptoms no operative procedures within the pelvis, not even the passage of a sound, can be safely undertaken. The influence of this teaching has been widely felt, and as far as it relates to the last stage, the chronic stage, of pelvic cellulitis, it is unquestionably good doctrine. But I have so often met with cases presenting hardness and tenderness of the vaginal roof without previous history of acute cellulitis, which bear with impunity any amount of operative interference, that I have been led to believe that a radical difference exists between the two classes of cases, and to seek out the cause for such a difference. I have found that the latter class of cases includes the larger number of those which have their origin in injuries received in labor, and particularly in lacerations of the cervix, especially when such lacerations have been of long standing.

In such cases I have found the womb to be of greater than normal size, and low down in the pelvis, so that the cervix is supported by the posterior vaginal wall. The lips are everted, exposing the mucous membrane of the cervical canal, which is in a state of inflammation and studded with enlarged Nabothian follicles. On either side of the cervix, extending outward, are irregular hard masses which are tender to the touch. These masses do not disappear upon pressure. The womb is not fixed in its abnormally low position, but may be easily elevated or moved from side to side. When the womb is raised upon the finger, so as to lift it from the posterior vaginal wall and restore it to its normal elevation, and maintained in that position for ten or fifteen minutes, I have so repeatedly felt these hard masses soften and diminish in size under my touch that I consider it impossible for me to have been deceived in regard to the occurrence of this phenomena. Basing a plan of treatment upon this observation, I have for a long time been in the habit of making use of a mechanical device for keeping the womb thus elevated, and under such treatment I have been amazed at the rapidity with which the tenderness and the hardness have disappeared. I have known them, even when well marked, to be entirely gone at the end of forty-eight hours, and their disappearance is quickly followed by the abatement of all pelvic symptoms, such as pain, bearing-down, frequent micturition, leucorrhœa and irregular menstruation. The patient rapidly gains flesh and strength, nervous symptoms are relieved, and, after a week or ten days of such treatment, she feels like a new creature. If examined at the end of that time the hardness and tenderness are sought for in vain, the womb is notably diminished in size and the patient feels so well that it is difficult to convince her of the necessity of operative treatment for the relief of the injury which formed the starting point of her disease. This is no fancy picture, but a description of what I have seen over and over again, and it will be corroborated by gentlemen who have had an opportunity of watching my practice. I have under my care at the present time, at the Benevolent Home, in this city, by the courtesy of the attending physician, Dr. N. O. Harris, two patients of this class, who form striking illustrations of the results of this mode of treatment. Three weeks ago they were anæmic, nervous, suffering, sleepless, bed-ridden creatures, a burden to themselves and to all about them. To-day they are free from pain, they sleep and eat normally, are rapidly gaining flesh and express themselves as feeling well in all respects. In neither case is there a history of any acute pelvic inflammation. The one has a double laceration of the cervix, with marked eversion and a laceration of the perineum to the sphincter. The other has an equal degree of perineal laceration and a prolapse of the urethra. In both cases these injuries still remain unrepaired.

Let us contrast with these a case in which the disease has originated in an acute pelvic cellulitis, and has progressed through the stages of infiltration and of solidification until it comes under the notice of the physician in the latter stage. (As in Case iii.) Here also the womb is found heavy, tender and low in the pelvis. On each side are felt irregular, hard masses, extending from the cervix outward, along the broad ligaments, tender to the touch. But if any attempt be made to raise such a womb, it will be found to be more or less immovable, and any endeavor to alter its position, even temporarily, will result in an increase of pain and probably a renewal of the acute stage of inflammation. Under favorable treatment the hard masses of plastic exudation will soften down and disappear in a period varying from two to four months. The subjective symptoms will be relieved only *pari passu* with the disappearance of the exudation in the cellular tissue. My experience has led me to believe that this condition exists in only a small minority of the cases of pelvic tenderness and hardness which come under the notice of the physician. These are undoubtedly cases of true chronic pelvic cellulitis.

On the other hand, I believe that the class first alluded to are not cases of pelvic cellulitis, but of some condition entirely different. My reason for this belief is found chiefly in the rapidity with which the physical phenomena disappear under appropriate treatment. It is inconceivable that a chronic inflammatory deposit, which has existed for months and perhaps for years, should be appreciably diminished in a few minutes, or that it should be entirely taken up and carried off by the blood-vessels and absorbents in forty-eight hours. No solid tissue undergoes such rapid changes as that. The only theory which appears to me to accord with these facts is that the hard masses on either side of the cervix are accumulations of venous blood within the distended venous sinuses, which are so abundant and so large in this locality. Such accumulations could be caused only by some obstruction to the venous current at some point within the pelvis.

A study of the anatomical distribution of the veins of the pelvis, and of the inevitable results of uterine congestion upon the pelvic circulation, lends confirmation to this theory. The uterine vein passes downward along the lateral border of the uterus, receiving at various points horizontal branches from that organ. At a point about midway between the external and internal os it passes outward through the cellular tissue to the wall of the bony pelvis and thence upward toward the heart. In its passage through the cellular tissue of the broad ligament it loses the characteristics of a vein and develops into a series of enormous distensible sinuses. Throughout its course it is entirely free from valves. Any obstruction at any point in the uterine vein must, therefore, necessarily result in damming up the blood behind the obstruction and causing a distention of that vein.

Whenever any injury of any kind to the pelvic organ takes place during labor, and remains unrepaired, involution is interfered with. The womb fails to return to its normal size and is therefore of greater than normal weight. Consequently the natural equilibrium between the uterine supports and the weight to be supported is destroyed, and the organ sinks below its normal level in the pelvis. Savage has shown that when the womb sinks below its normal level traction is first exerted upon the cellular tissue of the broad ligaments, "especially where it surrounds and accompanies the uterine blood-vessels."[1] Traction upon an elastic tissue necessarily involves traction upon the blood-vessels within that tissue, and traction upon an elastic tube, like a blood-vessel, means diminution of its calibre and obstruction of the flow of blood through it. Hence, when the womb, in a state of sub-involution from parturient injury, sinks below its normal level from increased weight, a condition exists which can give rise to obstruction of the uterine vein and its consequent distention. When the womb is raised the obstruction is removed and the distention of the venous sinuses is at once relieved.

I have never been so situated, much to my regret, as to be able to corroborate this theory by systematic *post mortem* study. I have sought in vain for the observations of others in this field until the recent appearance of an article by Henry C. Coe, Pathologist to the New York State Woman's Hospital.[2] The writer

---

[1]The Surgery, Surgical Pathology and Surgical Anatomy of the Female Pelvic Organs. New York, 1880. Plates XVIII and XIX.

[2] "Exaggerated Importance of Minor Pelvic Inflammations." New York Medical Journal, May 15, 1886.

of this article concludes, from the examination of a large number of subjects who, during life, presented the hard and tender masses on one or both sides of the cervix, which are commonly regarded as evidence of the existence of chronic pelvic cellulitis, that no appearances are found after death to correspond with the condition existing during life. No such masses in the pelvic cellular tissue can be detected. He says: "In by far the greater number of cases where a well-marked laceration of the cervix was present, there was absolutely no induration whatever in the broad ligaments. This 'thickening,' 'increased tension,' or whatever it is, which we find at the examining table and promptly enter in our case-books as 'cellulitis,' frequently vanishes after death, when the natural tension of the tissues has disappeared." "I would add briefly that in only a single case have I felt satisfied that I found well-marked remains of a cellulitis in a case of laceration of the cervix." There is no tissue of the body, either normal or abnormal, which can so completely disappear in a few hours after death as to leave no trace of its existence. But if, as I believe, the masses in question are simply accumulations of venous blood, it can readily be seen how it may become impossible to discover their existence in the dead-house. As far as they go, therefore, the results of *post mortem* investigation confirm my theory.

The treatment based upon these views is simple and obvious. It consists in maintaining the womb at its normal level by some mechanical device. The one which I have been led to adopt as preferable to all others is that which I have called the supporting tampon. It consists of a ring of four pledgets of cotton placed around the cervix, so graduated in size as to bring the cervix as nearly as possible into the normal axis, and a larger underlying pad of the same material, of such size as to raise the womb as a whole to its normal level. Any other device, which accomplishes the same purpose without producing undue pressure at any point, would be equally good. I use the supporting tampon because, after long experience, I have found it most practicable and most satisfactory.

By this mode of treatment patients are sometimes permanently cured. When the parturient injury is slight, the good results of treatment sometimes persist, and the symptoms do not return. Such results, however, form the exception. In the majority of cases before a permanent cure can be effected, the parturient injury must be repaired. Otherwise the womb will again sink below its normal level as soon as the mechanical support is removed and the original condition will be reproduced. It may be asked, "What, then, is gained by this treatment? Why not operate at first?" My answer is that such patients are not usually in a condition for operation when first seen by the physician. They are debilitated, nervous, depressed, sleepless, dyspeptic, and require "building up" before any operation can be successfully undertaken. If the womb in such cases be maintained at its normal level for a short time, the nervous symptoms subside, the general health is restored, the *morale* is improved and confidence is inspired, all of which are valuable factors in the success of an operation.

My object in writing this paper is to submit the following propositions:

1. Acute pelvic cellulitis in the stage of infiltration may frequently be aborted by aspiration.

2. Chronic pelvic cellulitis rarely, if ever, exists except as a sequence of a previous acute pelvic cellulitis.

3. Hardness and tenderness in the broad ligaments, as a result of pelvic venous engorgement, are commonly mistaken for chronic pelvic cellulitis.

4. The treatment of such engorgement by raising the womb in the pelvis relieves the constitutional as well as the local symptoms and places the patient in a suitable condition for a radical operation more speedily than the methods of treatment commonly in vogue.

